# Ultrasound-Guided Percutaneous Aspiration for the Treatment of Breast Abscess at a Tertiary Care Center in the Developing World

**DOI:** 10.7759/cureus.30865

**Published:** 2022-10-30

**Authors:** Shaista Afzal, Ahmad Bashir, Hania Shahzad, Imrana Masroor, Abida K Sattar

**Affiliations:** 1 Radiology, Aga Khan University Hospital, Karachi, PAK; 2 Urology, The Aga Khan University Hospital, Karachi, Karachi, PAK; 3 Surgery, Aga Khan University, Karachi, PAK; 4 Surgery, Aga Khan University Hospital, Karachi, PAK

**Keywords:** breast abscess, surgical debridement, ultrasound-guided fine-needle aspiration, benign breast lesion, percutaneous aspiration

## Abstract

Purpose: Percutaneous ultrasound (US)-guided aspiration is the first line of management for breast abscess. Our study aimed to look at the success of US-guided percutaneous drainage in managing breast abscesses at a tertiary care center and additionally to look for any correlation between US features and failure rate. Methods: A retrospective review of the radiology database at a tertiary care hospital in Pakistan was done to identify 54 patients through non-probability convenience sampling who underwent a US-guided percutaneous aspiration with laboratory confirmation of abscess. A treatment course was observed for the development of complications or failure of treatment. A chi-square test was performed to correlate US features and patient characteristics with outcomes of treatment (p<0.05). Fisher’s exact test was applied to evaluate the success of aspiration in small versus large abscesses, and in lactating versus non-lactating patients.

Results: 75% of all women were successfully able to avoid surgery. Specifically, 80.6% of all lactating women and 66.7 % of non-lactating women with breast abscesses were successfully managed with US-guided percutaneous aspiration. Across a variety of parameters measured, including pathological and etiological factors, as well as features on imaging, no significant association was established between the variables and the failure of the intervention.

Conclusion: Low morbidity and high patient satisfaction rates make percutaneous aspiration preferable to surgical intervention as a first-line treatment of breast abscess. Early use of antibiotics is recommended as an adjunct to drainage.

## Introduction

Breast abscesses are commonly encountered in routine practice by healthcare providers ranging from primary care physicians to breast surgeons. Early recognition and prompt treatment are imperative to minimize morbidity. It is therefore essential that all potential providers are well-aware of contemporary approaches or best practices for the management of breast abscesses.

The treatment involves drainage of the purulent material along with antibiotic coverage. Conventionally, surgical incision and drainage were common practice [1,2]. In recent years, percutaneous needle aspiration, with or without ultrasound (US) guidance has superseded surgical drainage [3]. Current recommendations include image-guided aspirations as the first line with surgical drainage being reserved for non-resolving abscesses or those presenting with necrotizing signs [4]. Unfortunately, conventional surgical procedures like incision and drainage are still commonly practiced in several parts of the world including Pakistan.

While US-guided aspiration of breast abscesses is a common practice worldwide, it is rarely practiced in the developing world. To date, there are limited studies on this practice in our settings. We aimed to study the frequency of US-guided aspiration of breast abscess in our setting and to evaluate the different factors associated with it.

## Materials and methods

Data sources

We searched PubMed and Google Scholar for all the previous work on the topic using text words and medical subject headings (MeSH) terms “Benign Breast Lesion”, “Percutaneous Aspiration”, “Ultrasound-guided Fine-Needle Aspiration”, and “Surgical debridement”. Our search included meta-analyses, randomized controlled trials, clinical trials, and reviews till 30th June 2021. Based on the title and abstracts, 30 relevant studies were found that were used to help us develop our research methodology.

Methods

**Figure 1:**
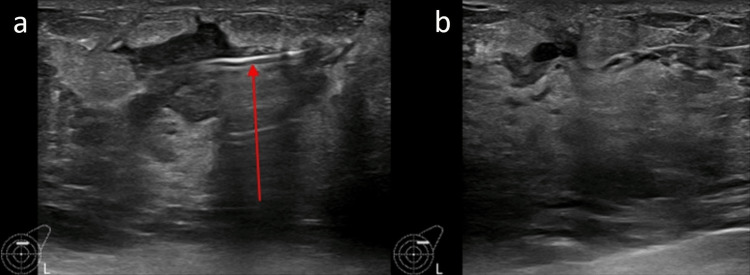
(a) Ultrasound image of the aspiration of abscess in the left upper and outer quadrant, the needle tip (arrow) is seen in the center of the abscess; (b) Ultrasound image post successful aspiration where no residual collection is seen

After approval from the institutional ethics review committee, we reviewed the radiology database for patients referred for breast abscess aspiration from 1st January 2016 to 31st December 2018. Using non-probability convenience sampling, we included patients that underwent US-guided breast abscess aspiration and a microbiology/pathology specimen confirmation of an abscess (Figure 1). Patients with incomplete records were excluded. Records of 54 patients were reviewed. The success of percutaneous US-guided aspiration was defined as the resolution of the abscess without the need for operative intervention, and failure was defined as no resolution eventually requiring surgical intervention [5]. More than one US-guided aspiration performed within 30 days was taken as serial aspiration. A new infection was considered if the abscess recurs after clinical and/or imaging documentation of complete resolution. The variables collected included patient demographics, lactational status, imaging features of the abscess such as size, site, loculation, size of residual abscess cavity after aspiration, number of aspirations performed, size of residual abscess on follow-up, results of gram stain, culture, and sensitivity and need for incision and drainage (I&D). Poor parameters were defined as ones that warranted I&D.

Analysis

Data was entered and analyzed on Statistical Package for Social Sciences (SPSS) version 20.0 (IBM Corp., Armonk, NY, USA). Means and standard deviation were computed for quantitative variables like age. Proportions were reported for qualitative variables. A chi-square test was used to evaluate the association between imaging features and failure of the procedure. A p-value<0.05 was considered to be significant. Due to the small sample size, Fisher’s exact test was applied to evaluate the success of aspiration in small versus large abscesses, and in lactating versus non-lactating patients.

## Results

All 54 patients in this study were females. Among the age groups, most of the cases were seen in those aged 20 to 29 years (see Table 1). The most common presenting symptom was pain (n=41, 76%), followed by swelling (n=40,74%), fever (n=22, 41%), and redness (n=12, 22%). Only one had an ongoing spontaneous discharge. The relationship between all these parameters and the need for surgical drainage is displayed in Table 2. No parameter was seen to have a significant p-value.

**Table 1:** Demographic parameters and etiology of patients with breast abscess SLE: Systemic lupus erythematosus

Parameters	Frequency N (%)
Age (years)	
20-29	25 (46)
30-39	24 (44)
40-49	4 (8)
>50	1 (2)
Comorbidities	
Diabetes mellitus	2 (4)
Hypertension	3 (6)
Hypothyroidism	1 (2)
SLE	1 (2)
Etiology of breast abscess	
Lactational	36 (67)
Non-lactational	18 (33)

**Table 2:** Predictors of success and failure of ultrasound-guided aspirations Significant = p-value≤0.05; the chi-square test was used

Predictors		Total N (%)	Need for Operative Intervention		
			Successfully managed with aspirations N (%)	Failed percutaneous aspiration warranting I&D N (%)	P-value
Etiology	Lactational	36 (66.7)	29 (80.6)	7 (19.4)	N 0.319
	Non- lactational	18 (33.3)	12 (66.7)	6 (33.3)	
Duration of Symptoms	<5 days	12 (22.2)	9 (75)	3 (25)	1.000
	>5 days	42 (77.8)	32 (76.2)	10 (23.8)	
Number of Quadrants	1	43 (79.6)	34 (79.1)	9 (20.9)	0.499
	2	9 (16.7)	6 (66.7)	3 (33.3)	
	3	2 (3.7)	1 (50)	1 (50)	
Number of Aspirations	Single	30 (55.6)	23 (76.7)	7 (23.3)	1.000
	Multiple aspirations (>1)	24 (44.4)	18 (75)	6 (25)	
Size of Abscess	<3cm	9 (16.7)	7 (77.8)	2 (22.2)	1.000
	>3cm	45 (83.3)	34 (75.6)	11 (24.4)	
Number of Abscesses	Single	39 (72.2)	29 (74.4)	10 (25.6)	1.000
	Multiple	15 (27.8)	12 (80)	3 (20)	
Presence of Fistula	Yes	4 (7.4)	3 (75)	1 (25)	1.000
	No	50 (92.6)	38 (76)	12 (24)	
Septations	<3	20 (37)	17 (85)	3 (15)	0.87
	>3	6 (11.1)	6 (100)	0 (0)	
	None	28 (51.9)	18 (64.3)	10 (35.7)	
Use of Antibiotic	Yes	38 (70.4)	29 (76.3)	9 (23.7)	1.000
	No	16 (29.6)	12 (75)	4 (25)	
Duration of Antibiotic Use	>7 days	28 (75.7)	20 (71.4)	8 (28.6)	0.159
	<7 days	9 (24.3)	9 (100)	0 (0)	
Microbiology Culture	Staphylococcus aureus	35 (64.8)	29 (82.9)	6 (17.1)	0.178
	Staph species (not aureus)	1 (1.9)	1 (100)	0 (0)	
	*Corynebacterium* spp	2 (3.7)	2 (100)	0 (0)	
	Streptococcus Group D	1 (1.9)	1 (50)	1 (50)	
	Klebsiella pneumoniae	1 (1.9)	0 (0)	1 (100)	
	No Growth	13 (24.1)	9 (69.2)	4 (30.8)	

## Discussion

Patients in our study who presented with a breast abscess showed that most of them can be effectively managed by US-guided aspirations. We did not identify any US imaging features that could predict the success or failure of US-guided percutaneous aspiration.

Breast abscesses are a common finding in puerperal women [6]. This may be due to predisposing factors such as mastitis and the proliferation of glandular tissue in the breast which commonly occurs during lactation [7-9]. A majority of patients in our study had lactation-associated breast abscesses which are consistent with previous reports [6].

For breast abscesses showing non-resolution after the first aspiration attempt, repeated US-guided aspirations can be done [7]. The traditional management of lactational breast abscesses has been I&D in both high and low-income countries [10]. However, recent evidence suggests good outcomes with percutaneous aspiration with or without US guidance. A recent study reported success rates of 96% with US-guided percutaneous aspiration regardless of abscess size [11]. Similarly, data published in 2021 from Cameroon showed that with repeated aspirations, there are success rates of 24% with a single aspiration, 32% with two, and 46% with three aspirations [12]. We successfully managed with US-guided percutaneous aspirations in 75% of our cohort, despite late presentation possibly due to the cultural stigma attached to breast diseases [13].

Several studies have reported shorter resolution time with needle aspiration compared with I&D and less scar formation, though there was a higher failure rate in the aspiration group compared to open drainage [7, 14-16]. Despite the potential need for repeat intervention, patients may prefer minimally invasive percutaneous aspirations due to early resumption of breastfeeding, improved cosmesis, less pain, and no hospitalization [11,15-16]. Patients who undergo I&D may develop psychological anxiety due to post‑operative scar formation and deformity of the breast, with one report showing patient dissatisfaction rates of 70% [17]. In addition to this, these patients need daily dressing of the abscess cavity which is painful and also leads to an extra financial burden.

Our study did not identify any imaging feature that could predict the success or failure of the aspiration. Prior studies have reported an increased likelihood of multiple aspirations and worse outcomes of breast abscess in patients with a history of smoking and nipple rings [18]. These two factors were not reported in our data. Though previous studies suggest that patients who present within five days of symptom onset have higher success rates compared to those presenting after five to 10 days [19,20] and that the likelihood of success with a single aspiration may be higher for small abscesses [21]. The majority of our patients presented after five days of symptoms with abscesses that were usually larger than 3cms. Yet, they had very high success rates of 75% with percutaneous drainage.

Studies have suggested a higher failure rate for abscesses treated with aspiration in the presence of skin necrosis, centrally located abscess, multi-loculation, and non-lactational abscess, however, the exact value of their predictive nature is not well established [22]. None of our patients in the US-guided aspiration cohort had necrosis as it is likely that such patients were taken for surgical drainage. Not much difference in percentage was found between the lactational and non-lactational groups.

The most common organism causing breast abscess in our study was *Staphylococcus aureus* (65%), consistent with previous reports [23-25], though methicillin-resistant *S. aureus *(MRSA) was not detected in any of our samples, unlike other reports [26]. Thus, early recognition and prompt initiation of antibiotic therapy against *S. aureus* are important.

Limitations

Our data was limited and would be helped by including a comparison with patients that underwent upfront incision and drainage based on the clinician’s judgment. Moreover, the sample size was small and the nature of the study was retrospective. Furthermore, data on where these patients present within the hospital (emergency rooms, general surgery clinics, family medicine clinics) also determines the choice of care provided and our study did not take into account patients other than those from breast surgery clinics.

## Conclusions

Seventy-five percent of our breast abscess patients selected and referred for US-guided percutaneous abscess aspiration were able to avoid surgery with the majority requiring only a single aspiration. Lower morbidity and higher patient satisfaction rates with percutaneous aspiration make it an essential consideration in the treatment algorithm. Incision and drainage with its associated deformity and higher morbidity should be deferred in favor of the minimally invasive approach when possible. Though our study is limited by the small sample size and failed to identify factors predictive of failure of percutaneous aspiration treatment, it may be worth exploring in future studies. Antibiotics, especially with coverage for* S. aureus*, must be commenced early.
